# *LRRFIP1*, an epigenetically regulated gene, is a prognostic biomarker and predicts malignant phenotypes of glioma

**DOI:** 10.1111/cns.13817

**Published:** 2022-03-26

**Authors:** Wenping Ma, Zhaoshi Bao, Zenghui Qian, Kenan Zhang, Wenhua Fan, Jianbao Xu, Changyuan Ren, Ying Zhang, Tao Jiang

**Affiliations:** ^1^ Department of Molecular Neuropathology Beijing Neurosurgical Institute Capital Medical University Beijing China; ^2^ Department of Neurosurgery Beijing Tiantan Hospital Capital Medical University Beijing China; ^3^ Center of Brain Tumor Beijing Institute for Brain Disorders Beijing China; ^4^ China National Clinical Research Center for Neurological Diseases Beijing China; ^5^ Chinese Glioma Genome Atlas Network (CGGA) and Asian Glioma Genome Atlas Network (AGGA) Beijing China; ^6^ The Second Affiliated Hospital of Harbin Medical University Harbin China; ^7^ Sanbo Brain Hospital Capital Medical University Beijing China

**Keywords:** biomarker, DNA methylation, glioma, LRRFIP1, prognosis

## Abstract

**Aims:**

Glioblastoma (GBM) is the most common malignant brain tumor with an adverse prognosis in the central nervous system. Traditional histopathological diagnosis accompanied by subjective deviations cannot accurately reflect tumor characteristics for clinical guidance. DNA methylation plays a critical role in GBM genesis. The focus of this project was to identify an effective methylation point for the classification of gliomas, the interactions between DNA methylation and potential epigenetic targeted therapies for clinical treatments.

**Methods:**

Three online (TCGA, CGGA, and REMBRANDT) databases were employed in this study. T‐test, Venn analysis, univariate cox analysis, and Pearson's correlation analysis were adopted to screen significant prognostic methylation genes. Clinical samples were collected to determine the distributions of LRRFIP1 (Leucine Rich Repeat of Flightless‐1 Interacting Protein) protein by immunohistochemistry assay. Kaplan–Meier survival and Cox analysis were adopted to evaluate the prognostic value of *LRRFIP1*. Nomogram model was used to construct a prediction model. GO (Gene Ontology) and KEGG (Kyoto Encyclopedia of Genes and Genomes) pathway were performed to explore functions and related mechanisms of LRRFIP1 in gliomas.

**Results:**

Our results showed that 16 genes were negatively connected with their methylation level and correlated with clinical prognosis of GBM patients. Among them, *LRRFIP1* expression showed the highest correlation with its methylation level. *LRRFIP1* was highly expressed in WHO IV, mesenchymal, and IDH wild‐type subtype. *LRRFIP1* expression was an independent risk factor for OS (overall survival) in gliomas.

**Conclusion:**

*LRRFIP1* is an epigenetically regulated gene and a potential prognostic biomarker for glioma. Our research may be beneficial to evaluate clinical efficacy, assess the prognosis, and provide individualized treatment for gliomas.

## Graphical abstract

Based on TCGA, CGGA, and REMBRANDT databases, we screen a significant prognostic methylation gene of *LRRFIP1* for an independent risk factor in gliomas. The prediction value is helpful for clinical efficacy evaluation, prognosis judgment, and probable individualized treatment strategies for glioma.

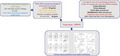

## 1 INTRODUCTION

GBM is the most common type of aggressive tumors with a median survival of 15 months, 5 years survival of 5.5%, accounting for 45.2% of primary brain malignancy.^1^, ^2^, ^3^, ^4^ It is of great clinical significance to investigate prognostic marker of GBMs for further precision therapy.^5^ Recently, mutations of isocitrate dehydrogenase 1 (*IDH1*) and methylation of O (6)‐methylguanine DNA methyltransferase (*MGMT*) promoter have been identified as molecular classification according to different clinical outcomes.^6^, ^7^ Patients with *IDH1* mutation were associated with higher OS than *IDH1* wild‐type patients.^8^ Patients with *MGMT* promoter methylation will benefit from Temozolomide (TMZ) treatments, and patients with lower MGMT protein have better prognosis.^9^ DNA methylation is an epigenetic mechanism involving regulating gene expression by recruiting proteins involved in gene repression or by inhibiting the binding of transcription factor to DNA in the mammalian genome.^10^ DNA methylation biomarkers with independent prognosis value have been rarely reported. Despite the rapid development of new drugs, the discovery of accurate biomarkers is still being explored.^11^ Therefore, the identification of novel evaluable biomarkers associated with DNA methylation in GBMs is urgently needed. In this work, TCGA RNA sequencing dataset, TCGA DNA methylation dataset (Illumina Human Methylation 27K and 450K), and CGGA RNA sequencing dataset were analyzed, and *LRRFIP1* was screened out as a potential prognostic factor. LRRFIP1 was also named as GCF2,^12^ FLAP1,^13^ and TRIP^14^, according to its differential functional splicing isoforms. Due to a variety of splicing isoforms, LRRFIP1 was involved in a wide range of biological functions, both in the nucleus and cytoplasm. Therefore, dysregulation of LRRFIP1 is critical in infections,^15^ autoimmune diseases,^16^ neurological,^17^ and cancers.^18^ It has been identified that LRRFIP1 plays a critical role in continuous growth, epithelial–mesenchymal transition (EMT), invasion, metastasis, and resistance to anti‐tumor drugs in cancers. However, the methylation and expression status of *LRRFIP1*, especially the function in glioma biology, are still unknown. In our study, we focus the potential prognostic value of LRRFIP1, giving new insights into the methylation role of LRRFIP1 in glioma.

## 2 MATERIAL S AND METHODS

### 2.1 Data acquisition and processing

TCGA DNA methylation dataset (Illumina Human Methylation 27K), TCGA DNA methylation dataset (Illumina Human Methylation 450K), TCGA RNA microarray, and TCGA RNA sequencing (RNAseq) dataset were downloaded from The Cancer Genome Atlas (TCGA, http://cancergenome.nih.gov/), which were analyzed for further discovery. CGGA RNAseq dataset and CGGA 27K methylation dataset were downloaded from the Chinese Glioma Genome Atlas (CGGA, http://www.cgga.org.cn/). The Repository for Molecular Brain Neoplasia Data (REMBRANDT, http://caintegrator‐info.nci.nih.gov/REMBRANDT) was included for our validation analysis. Also, the *LRRFIP1* mRNA expression and DNA methylation data in cell lines were obtained from Cancer Cell Line Encyclopedia (CCLE, http://portals.btoadinstitute.org/ccle/home).

### 2.2 Prognostic analysis

Kaplan–Meier survival curve and log‐rank test were used to evaluate the prognosis of methylation and expression level of *LRRFIP1* by R package “survminer.”

### 2.3 Univariate and multivariate cox proportional hazard models

Univariate and multivariate Cox proportional hazard models were estimated the prognostic value of *LRRFIP1* and other clinical related features (gender, age at diagnosis, WHO grade, *IDH* mutation status, 1p/19q codeletion status, *MGMT* promotor status, *EGFR* amplification, radiotherapy, and chemotherapy) by R package “survival.”

### 2.4 Nomogram construction and prediction

All patients with survival information from the CGGA, TCGA, and Rembrandt databases were collected to establish the nomogram. *LRRFIP1* expression and other clinical factors such as grade, 1p/19q status, chemotherapy, age, *MGMT* status, and radiotherapy were established using Cox regression in those databases. Calibration curves for different years were constructed to anticipate the total score for clinical risk features.

### 2.5 GO and KEGG enrichment analysis

The mRNA sequencing data were obtained from CGGA RNAseq and TCGA RNAseq database. *LRRFIP1*‐related genes were selected (*R* ≥0.5 & *p* < 0.05) according to Pearson's correlation analysis. DAVID (The Database for Annotation, Visualization and Integrated Discovery, https://david.ncifcrf.gov/) was used to analyze GO and KEGG enrichment analysis of these related genes.

### 2.6 Immunohistochemistry assay

The routine preparation and staining of paraffin sections were made as previously described.^3^ The protein evaluation was finished independently by two pathologists with the method as following. A: staining intensity (the average score of 3 different fields), the scores were as follows: negative staining =0 point; weakly positive staining =1 point; positive staining but with light brown background =2 points; positive staining without background =3 points. B: staining intensity: positive area =0%, designed 0 point; positive area =1%–25%, designed 1 point; positive area =26%–50%, designed 2 points; positive area =51%–75%, designed 3 points; positive area >75%, designed 4 points. C: the final scores were measured by multiplication of the values for A and B.

### 2.7 Statistical analysis

Pearson's correlation analysis was used to validate the correlation between *LRRFIP1* mRNA expression and methylation levels in glioblastoma. Student t‐test analysis was used to determine differences between the two groups. The statistically significant difference was considered when *p* < 0.05.

## 3 RESULTS

### 3.1 ***LRRFIP1*** **showed the highest correlation with its methylation level in glioblastoma**

**FIGURE 1:**
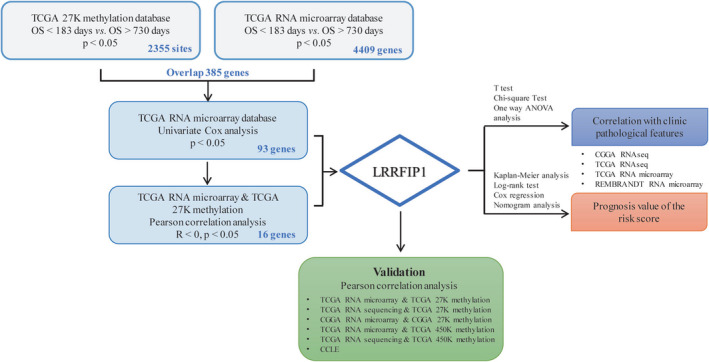
Workflow to identify LRRFIP1 and to show its potential prognostic value

According to the flowchart shown in Figure 1, we first set OS value less than 183 days as shorter OS group and that greater than 730 days as longer OS group. We applicated beta‐value statistic as metrics and identified 2355 differential sites in TCGA 27K methylation database. Meanwhile, we explored 4409 significantly differentially expressed genes between the shorter OS group and longer OS group in the TCGA RNA microarray database. We identified 385 genes via intersection. Then, we used univariate analysis to determine whether the expression of 385 genes are prognostic factors to OS of GBM patients. And 93 genes were statistically significant (*p* < 0.05, Table S1). Further, we used Pearson's correlation analysis to study the correlation between the methylation levels and expression levels of 93 genes. Finally, we distinguished 16 genes’ expression that showed negative correlation with their methylation levels (Table 1). Among them, *LRRFIP1* expression showed the highest correlation with its methylation level.

**TABLE 1:** Pearson's correlation analysis of the differential methylation gene with significant prognostic

Gene	Cox			Correlation		
	HR	95% CI	*p*	Location	*R*	*p*
*LRRFIP1*	1.647	1.155–2.349	5.88E−03	cg09037813	−0.407	1.58E−11
*C11orf58*	1.437	1.041–1.984	2.77E−02	cg09555217	−0.324	1.34E−07
*TCF15*	0.786	0.663–0.932	5.63E−03	cg22449114	−0.283	4.55E−06
*PRL*	0.615	0.449–0.843	2.54E−03	cg27541541	−0.247	7.04E−05
*TMEM9B*	1.260	1.006–1.579	4.46E−02	cg14205126	−0.243	1.04E−04
*PHTF1*	1.391	1.090–1.775	8.07E−03	cg21539243	−0.233	1.95E−04
*MOBKL1B*	1.262	1.020–1.561	3.18E−02	cg08434152	−0.225	3.02E−04
*STAM2*	1.356	1.015–1.811	3.92E−02	cg24904765	−0.214	6.13E−04
*CLDN12*	1.382	1.069–1.786	1.36E−02	cg02399449	−0.199	1.42E−03
				cg18967846	−0.197	1.64E−03
*ARNTL*	1.174	1.008–1.367	3.94E−02	cg13250711	−0.183	3.48E−03
*CYB5R1*	1.241	1.021–1.508	3.03E−02	cg18275051	−0.179	4.16E−03
*GPR65*	1.105	1.007–1.211	3.41E−02	cg15625636	−0.176	4.87E−03
*FSIP1*	1.273	1.086–1.492	2.89E−03	cg22936016	−0.173	7.92E−03
*AKAP12*	1.144	1.022–1.281	1.93E−02	cg12061236	−0.172	6.13E−03
*FECH*	1.417	1.049–1.914	2.31E−02	cg14532644	−0.150	1.68E−02
*GLIS1*	0.868	0.755–0.999	4.78E−02	cg21142398	−0.125	4.59E−02

### 3.2 Negative correlation between *LRRFIP1* expression and DNA methylation in gliomas

**FIGURE 2:**
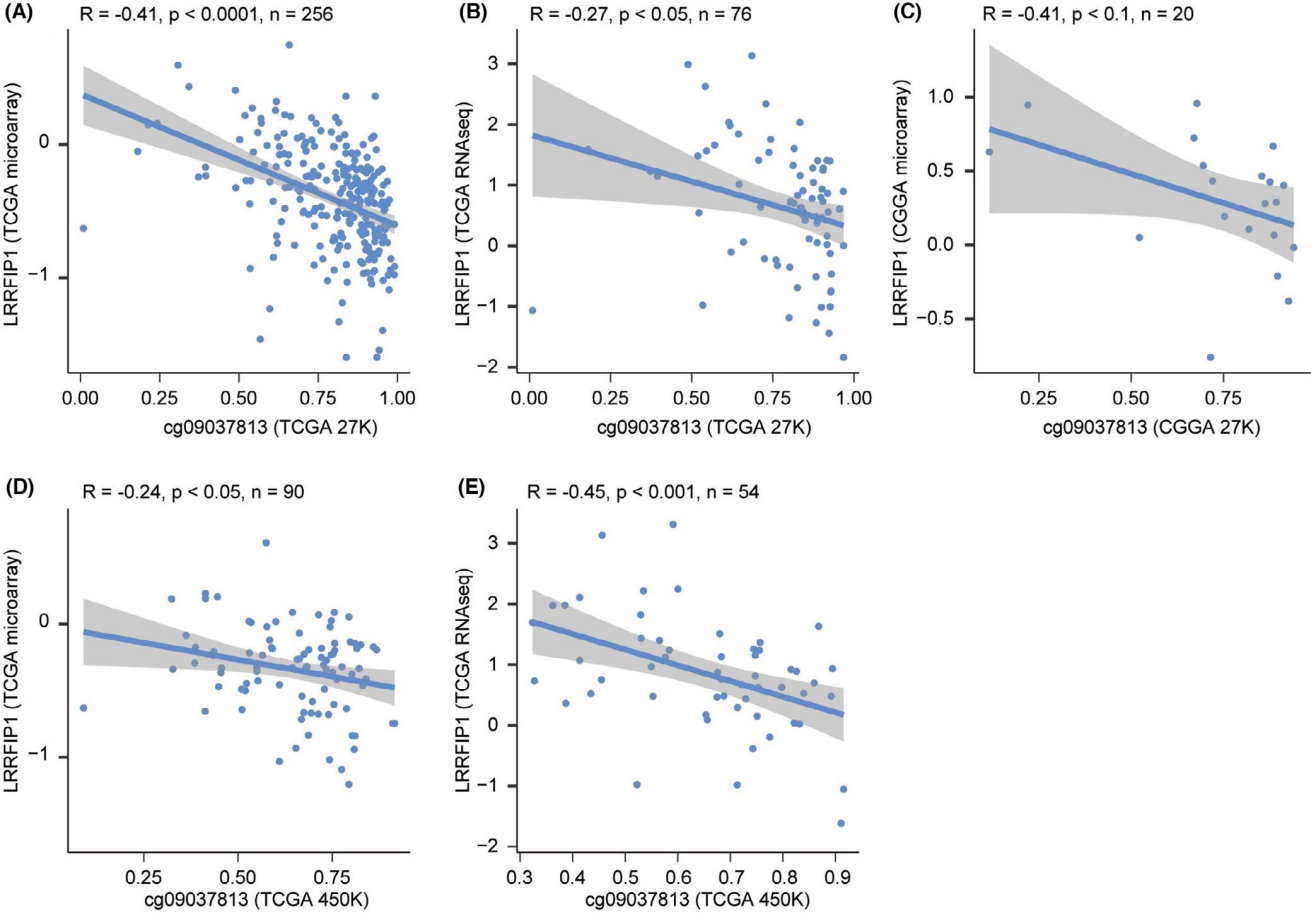
Validation of the correlation between the mRNA expression level of *LRRFIP1* and the methylation level in gliomas. (A, B) The mRNA expression level of *LRRFIP1* both in TCGA microarray database and in TCGA RNAseq database was negatively correlated with the methylation level in TCGA 27K methylation database (*R* = −0.41, *p* < 0.001, *n* = 256; *R* = −0.27, *p* < 0.05, *n* = 76, corresponding). (C) In CGGA 27K methylation database, the mRNA expression level of *LRRFIP1* was negatively correlated with the methylation level (*R* = −0.41, *p* < 0.1, *n* = 20). (D, E) The mRNA expression level of *LRRFIP1* both in TCGA microarray and in TCGA RNAseq database decreased with the increase in the methylation level of glioma in TCGA 450K methylation database (*R* = −0.24, *p* < 0.05, *n* = 90; *R* = −0.45, *p* < 0.001, *n* = 54, corresponding)

For validation, we first explored the correlation between the methylation level of *LRRFIP1* and the mRNA expression in GBM databases. mRNA expression of *LRRFIP1* was almost negatively correlated with methylation levels in gliomas, which was consistent in the discovery dataset (TCGA 27K methylation dataset, TCGA microarray, *n* = 256, *R* = −0.41, *p* < 0.0001; TCGA RNAseq, *n* = 76, *R* = −0.27, *p* < 0.05) and validation datasets (CGGA 27K methylation dataset, CGGA microarray, *n* = 20, *R* = −0.41, *p* < 0.1; TCGA 450K methylation dataset, TCGA microarray, *n* = 90, *R* = −0.24, *p* < 0.05; TCGA RNAseq, *n* = 54, *R* = −0.45, *p* < 0.001) (Figure 2A–E). In all cell lines, *LRRFIP1* expression is negatively correlated with DNA methylation level (A: *n* = 835, *R* = −0.39, *p* < 0.0001; B: *n* = 828, *R* = −0.42, *p* < 0.0001) (Figure S1A,B). In GBM cells, *LRRFIP1* expression has more high degree of negative correlation with DNA methylation level, behave as a larger value (C: *n* = 43, *R* = −0.45, *p* < 0.01; *n* = 42, *R* = −0.55, *p* < 0.001) (Figure S1C,D). Therefore, significant differences existed in the negative correlation between *LRRFIP1* expression and DNA methylation among glioma cell lines.

### 3.3 Low methylation of *LRRFIP1* predicts poor prognosis

**FIGURE 3:**
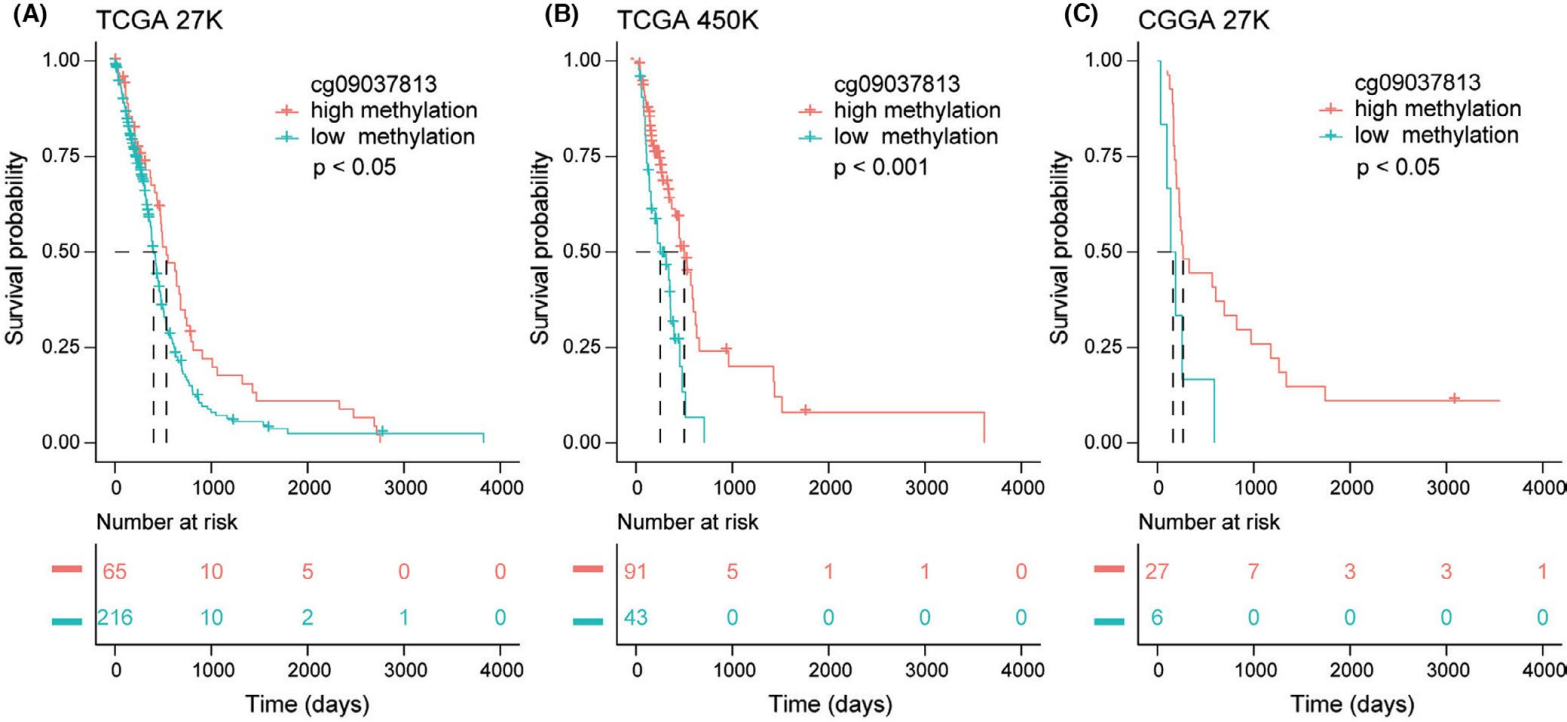
Prognostic significance of *LRRFIP1* methylation level in gliomas. (A‐C). Kaplan–Meier curves were used to estimate the methylation status of *LRRFIP1* with patient survival probability in TCGA 27K methylation database, TCGA 450K methylation database, and CGGA 27K methylation database. The methylation status of *LRRFIP1* was divided into two groups: high methylation and low methylation. The high methylation status of *LRRFIP1* has a longer survival probability than the low methylation status of *LRRFIP1* (A: *p* < 0.05; B: *p* < 0.001; C: *p* < 0.05)

Besides, we investigated the prognostic significance of *LRRFIP1* methylation level in TCGA 27K methylation dataset, TCGA 450K methylation dataset, and CGGA 27K methylation dataset. In TCGA 27K dataset, patients with high methylation level of *LRRFIP1* showed better outcomes than low methylation level (Figure 3A, *p* < 0.05). Moreover, these findings can be validated in the additional two datasets (Figure 3B, TCGA 450K methylation dataset, *p* < 0.001; Figure 3C, CGGA 27K methylation dataset, *p* < 0.05). These results indicated that low methylation of *LRRFIP1* predicts poor prognosis.

### 3.4 Distribution of *LRRFIP1* in clinical features in gliomas

**FIGURE 4:**
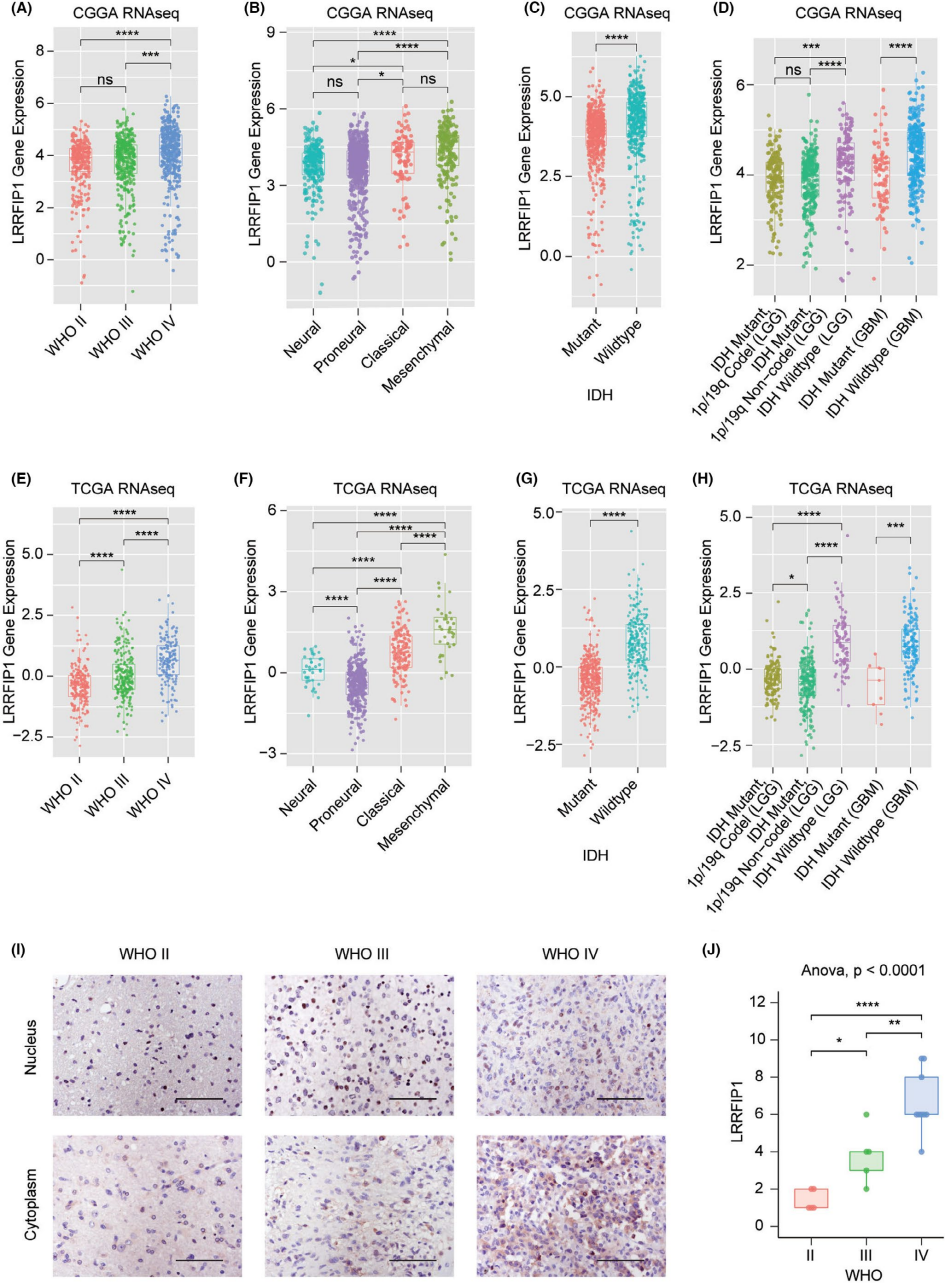
LRRFIP1 expression pattern in glioma patients. (A, E) *LRRFIP1* is enriched in high‐grade gliomas in CGGA RNAseq and TCGA RNAseq. (B, F) *LRRFIP1* is enriched in mesenchymal molecular subtype gliomas in CGGA RNAseq and TCGA RNAseq. (C, G) *LRRFIP1* is enriched in *IDH* wild‐type gliomas in CGGA RNAseq and TCGA RNAseq. (D, H) *LRRFIP1* expression is highest in *IDH* wildtype with GBM and lowest in *IDH* mutant combined with 1p/19q codeletion low grade gliomas in CGGA RNAseq and TCGA RNAseq. (I) LRRFIP1 expression in different grades of gliomas by IHC staining and (J) statistical analysis (II, *n* = 5; III, *n* = 5; IV, *n* = 9). Scale bar =100 μm. *, **, and ****, respectively, indicate *p* < 0.05, *p* < 0.01, and *p* < 0.0001

In order to understand the distribution of *LRRFIP1* mRNA expression in GBM, we explored the *LRRFIP1* mRNA expression in glioma's clinical features. As established in Figure 4A and E, *LRRFIP1* has the highest expression in WHO IV, both in CGGA and TCGA RNAseq databases. The *LRRFIP1* mRNA expression in WHO IV has statistical difference when compared with that in WHO II and WHO III, both in CGGA and TCGA RNAseq databases (Figure 4A and E). The expression of *LRRFIP1* was different among those subtypes of glioma in CGGA and TCGA RNAseq databases (Figure 4B and F). Since molecular diagnosis has gradually become a clinical application of precision medicine, we explored *LRRFIP1* expression in *IDH* status and 1p/19q codeletion. In CCGA and TCGA RNAseq databases, the expression of *LRRFIP1* in *IDH* wildtype is significantly higher than in *IDH* mutant (Figure 4C and G). Further, in CGGA RNAseq database, the expression of *LRRFIP1* in *IDH* mutant combined with 1p/19q codeletion LGG group has no statistical difference when compared with *IDH* mutant with 1p/19q non‐codeletion (Figure 4D), there was no obviously difference in TCGA RNAseq database (*p* < 0.05, Figure 4H). In LGG group, *IDH* mutant combined with 1p/19q codeletion has significant statistical difference when compared with *IDH* wildtype (*p* < 0.0001); *IDH* mutant combined with 1p/19q non‐codeletion also showed significant differences when compared with *IDH* wildtype (*p* < 0.0001) in CGGA and TCGA RNAseq databases (Figure 4D and H). In GBM groups, *IDH* mutant has significant statistical difference, as compared with *IDH* wildtype, both in CGGA (*p* < 0.0001, Figure 4D) and TCGA (*p* < 0.001, Figure 4H) RNAseq databases. Similar results about the distribution of *LRRFIP1* were presented when we employed REMBRANDT and TCGA microarray databases as validation (Figure S2). We also detected the protein level of LRRFIP1 in clinical glioma specimens, and positive expression of LRRFIP1 was located in cytoplasm and nucleus of tumor cells (Figure 4I). LRRFIP1 expression increased with tumor grade, especially concentrated in high‐grade gliomas (Figure 4I and J). Collectively, these results indicated that LRRFIP1 was correlated with clinical features of glioma.

### 3.5 *LRRFIP1* is an independent prognostic factor in glioma patients

**FIGURE 5:**
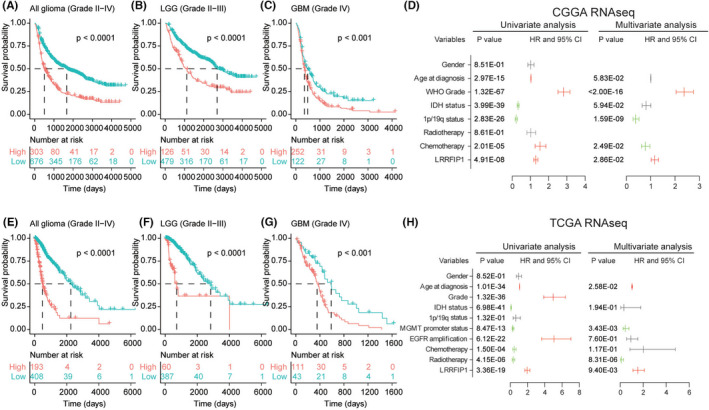
*LRRFIP1* mRNA expression was related to clinical outcomes in gliomas. (A‐C, E‐G). Kaplan–Meier analysis of the survival of all gliomas, LGG and GBM patients from the data of CGGA RNAseq and TCGA RNAseq. High expression of *LRRFIP1* was negatively associated with the OS of all gliomas, LGG and GBM. (D, H) Univariate and multivariate regression analyses of *LRRFIP1* expression level and other clinical features in CGGA RNAseq and TCGA RNAseq

To explore the prognostic value of *LRRFIP1*, we collected survival data from CGGA and TCGA RNAseq databases and investigated the correlation between *LRRFIP1* mRNA expression level and prognosis. The results demonstrated that high *LRRFIP1* expression was negatively correlated with glioma patients’ survival probability in all grades, LGG and GBM based on CGGA RNAseq database (Figure 5A–C, p < 0.001). As shown in Figure 5E–G, the elevated *LRRFIP1* expression was clinically correlated with unfavorable outcomes of glioma patients in all grades, LGG and GBM based on TCGA RNAseq database. We explored univariate and multivariate cox proportional hazard models to anticipate *LRRFIP1* prognosis for glioma patients in CGGA RNAseq database. The result showed that *LRRFIP1* was an independent risk factor (univariate, hazard ratio (HR) >1, *p* = 4.91E‐08; multivariate, HR >1, *p* = 2.86E‐02) (Figure 5D). In the TCGA RNAseq database, we acquired the *LRRFIP1* and other related clinical features, and *LRRFIP1* expression was also an independent risk factor (univariate, HR >1, *p* = 3.36E‐19; multivariate, HR >1, *p* = 9.40E‐03) (Figure 5H). Similar results were presented in the REMBRANDT dataset (univariate, HR >1, *p* = 1.98E‐07; multivariate, HR >1, *p* = 3.14E‐03) and TCGA microarray (univariate, HR >1, *p* = 5.03E‐04; multivariate, HR >1, *p* = 3.54E‐02) (Figure S3). These results suggested that *LRRFIP1* was an independent prognostic factor to predict the OS of glioma patients.

**FIGURE 6:**
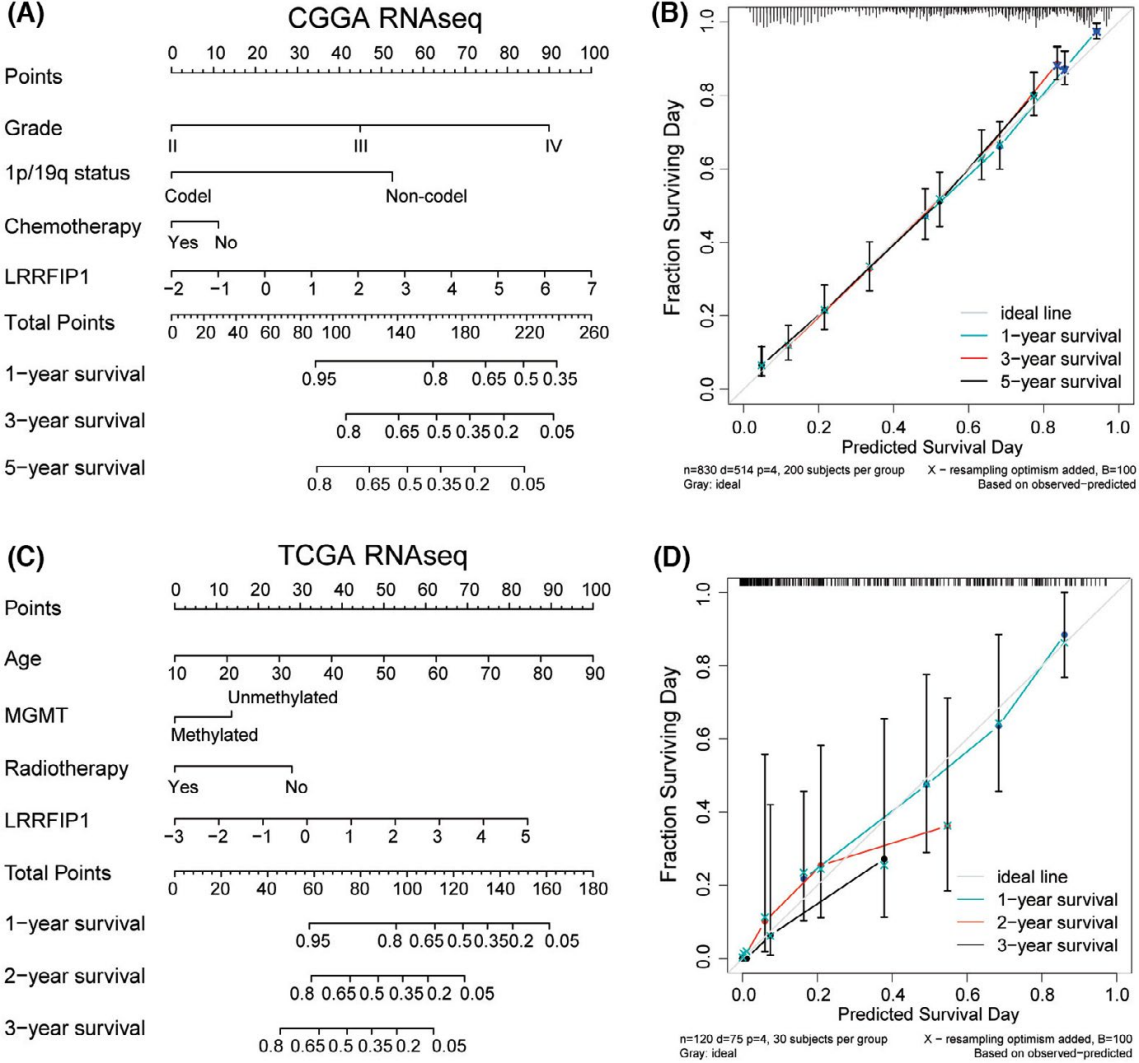
Construction and evaluation of the nomogram for predicting overall survival. (A) Nomogram for predicting 1, 3, or 5‐ year survival in glioma patients, based on the data from CGGA RNAseq. The top row shows the point value for each variable. Rows 2–5 indicate the variables included in the nomogram. Each variable corresponds to a point value were according to glioma's clinical characteristics. The sum of these values is located on the axis of total points, and downward the total points axis survival axes were drawn to determine the probability of 1‐, 3‐, or 5‐year survival. (B) Calibration curves for predicting patient survival at 1, 3, and 5 years in the dataset from CGGA RNAseq. (C) Nomogram for predicting 1, 2, or 3‐year survival in glioma patients, based on the data from TCGA RNAseq. (D) Calibration curves for predicting patient survival at 1, 2, and 3 years in the dataset from TCGA RNAseq

An effective nomogram model for OS was predicted by the significant factors. The predictive model was presented as a nomogram and is shown in CGGA RNAseq dataset (Figure 6A) and TCGA dataset (Figure 6C). The calibration plot for the probability of survival showed an optimal agreement between the prediction and observation in CGGA RNAseq (Figure 6B) and TCGA RNAseq datasets (Figure 6D), as well as in validation datasets (Figure S4). We show how the value of *LRRFIP1* can lead to better predictive models, and a deeper understanding of the function of methylation in gliomas.

### 3.6 **LRRFIP1** **may promote glioma progression**

**FIGURE 7:**
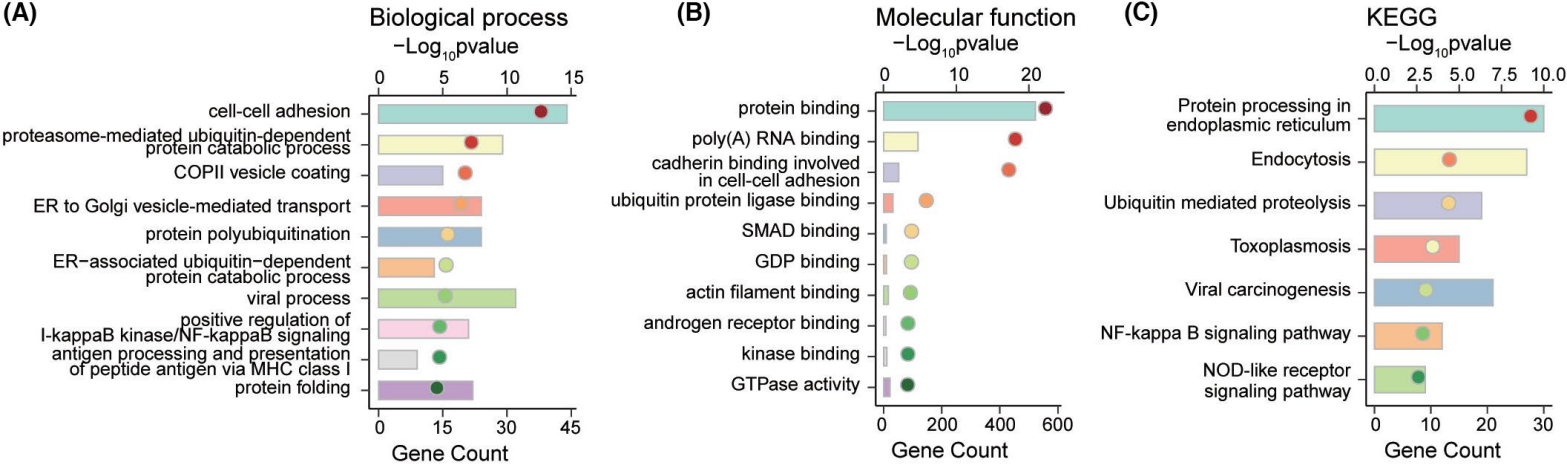
GO and KEGG enrichment analysis of *LRRFIP1*‐correlated genes in CGGA RNAseq database. (A) *LRRFIP1*‐associated biological process in gliomas. (B) *LRRFIP1*‐related molecular function in gliomas. (C) KEGG pathways regulated by LRRFIP1 in gliomas

A total of 862 *LRRFIP1*‐related genes (*R* ≥ 0.5, *p* < 0.05) in CGGA RNAseq database were selected into GO and KEGG enrichment analysis. The results of function analysis showed that the biological processes were mainly enriched in cell–cell adhesion, proteasome‐mediated ubiquitin‐dependent protein catabolic process, *etc*., (Figure 7A). Molecular function was mostly focused on protein binding, poly(A) RNA binding, *etc*., (Figure 7B). KEGG pathway analysis showed that LRRFIP1 were mainly related to protein processing in endoplasmic reticulum, endocytosis, *etc*., (Figure 7C). Other 598 *LRRFIP1*‐related genes (R ≥ 0.5, *p* < 0.05) in TCGA RNAseq database were also performed for the functional analysis. The biological processes were mainly enriched in extracellular matrix organization, signal transduction, *etc*., (Figure S5A). Molecular function was mostly focused on protease binding, protein binding, *etc*., (Figure S5B). KEGG pathway analysis showed that LRRFIP1 was mainly related to ECM‐receptor interaction, focal adhesion, cytokine–cytokine receptor interaction, *etc*., (Figure S5C). In summary, LRRFIP1 may play a vital role in glioma progression.

## 4 DISCUSSION

Molecular diagnostics has become an important basis for understanding the genetics and molecular biology, which can benefit for the personalized therapy of glioblastoma.^19^ Inter‐tumoral heterogeneity played an important role in GBM heterogeneity and orchestrated patients’ prognosis.^20^ LRRFIP1 significantly enriched in high‐grade gliomas in TCGA, CGGA, and REMBRANDT databases, our results indicated that LRRFIP1 may act as the malignancy characters in glioma and closely relate to clinical outcomes.

Among the numerous biomarkers, only *IDH* mutations, *MGMT* promoter methylation, and 1p19q codeletion are being routinely used in clinic diagnosis for glioma patients,^21^ while other biomarkers are still in observation phage in clinical trials. More clinical trials data should be provided to personalized therapeutic strategies for GBM patients with least toxicity and better outcomes. Therefore, it is urgent to identify an independent prognostic biomarker to apply precise treatment for patients. DNA methylation orchestrates a vital role in tumorigenesis and tumor development via epigenetic regulation. DNA methylation may act as one of the mostly potential prognostic and predictive value for GBM.^22^, ^23^ DNA methylation patterns were associated with the mutations in *IDH1* or *IDH2* in lower grade gliomas, and mutations in histone 3 in pediatric high‐grade gliomas.^24^ Furthermore, *MGMT* silencing by promoter methylation in adult glioblastoma is a predictive biomarker for benefit from alkylating agent chemotherapy.^7^, ^9^

In order to screen methylation genes and explore their prognostic values in gliomas, we screened out a total of 4409 genes from TCGA RNA microarray database and 2355 sites from TCGA 27K methylation database by setting OS <183 days vs. OS 730 days. After univariate cox analysis and Pearson's correlation analysis, we identified *LRRFIP1* as a high level of methylation among those differential genes. Our study revealed that the mRNA level of *LRRFIP1* was negative correlated with its’ DNA methylation in GBM. Patients with low methylation level of *LRRFIP1* correlated with worse prognosis in all gliomas. *LRRFIP1* expression levels enriched in high‐grade gliomas comply with malignancy character. In *IDH* mutant combined with 1p/19q codeletion LGG group and *IDH* mutant with 1p/19q non‐codeletion LGG group, the expression of *LRRFIP1* was no significant difference in CGGA RNAseq database and no obviously difference in TCGA RNAseq database. High *LRRFIP1* expression indicated worse prognosis in all gliomas, LGG and GBM. Such expression was more pronounced in high‐grade gliomas. COX analysis verified that *LRRFIP1* acts as an independent prognosis factor in gliomas. Nomogram models were also performed to identify *LRRFIP1* prognostic value in gliomas.^25^, ^26^, ^27^

LRRFIP1 was originally identified as a protein that interacts with Drosophila organizing embryogenesis and myogenesis.^28^ Also, LRRFIP1 was proved as a transcriptional repressor and a MyD88‐interacting protein,^29^ which localized in the cytoplasm and directly bind to GC‐rich dsDNA. Besides, LRRFIP1 can work as a co‐stimulator for signals from the cell surface, being involved in Wnt canonical pathway,^30^ integrin signaling pathway,^31^ or the nuclear receptor dependent pathway.^32^ *LRRFIP1* is reported as a direct target of miR‐21,^18^ suggesting that *LRRFIP1* gene could be involved in GBM response to chemotherapeutic agent. LRRFIP1 acted important functions such as cell proliferation, distant metastasis, and invasion in the development of many malignant tumors.^26^ LRRFIP1 was highly expressed in most primary human hepatocellular carcinoma (HCC) tissues and HCC cell lines. Knockdown of *LRRFIP1* in those cell lines by RNAi inhibited cell growth and promoted cell apoptosis.^33^ LRRFIP1 promoted colorectal cancer metastasis and liver invasion through RhoA activation.^31^ LRRFIP1 increased the EMT in pancreatic cancer through the Wnt/β‐catenin pathway.^34^ Nomograms have been proved LRRFIP1 as a more accurate prognostic prediction in cholangiocarcinoma and cervical cancer.^35^, ^36^ The GO and KEGG function analysis revealed that LRRFIP1 may play important roles in glioma progression. In summary, our findings disclosed that LRRFIP1 may serve as an important factor in drug selection and prognostic judgment of glioma patients.

## 5 CONCLUSIONS

In conclusion, LRRFIP1 could provide diagnostic or prognostic information for gliomas, possibly also act as a new therapeutic target in gliomas. The detailed understanding of LRRFIP1 by epigenetically regulation may uncover a new direction for anti‐glioma therapy.

## CONFLICT OF INTEREST

All authors declare no conflict of interest.

## 6 AUTHOR CONTRIBUTIONS

Wenping Ma involved in specimen collection, IHC assay, project guidance, and article writing. Zhaoshi Bao, Zenhui Qian, and Kenan Zhang edited the manuscript. Ying Zhang, Wenhua Fan, Jianbao Xu, and Changyuan Ren involved in data statistical analysis, and figures plot and charts organization. Ying Zhang and Tao Jiang involved in conception, supervision, and design of all manuscripts.

## ETHICAL APPROVAL

The research involving experiments on human subjects met the ethical standards of the Helsinki Declaration. The research was approved by the institutional review board of Beijing Tiantan Hospital, Capital Medical University, and all patients/relatives had provided written informed consent.

## 7 DATA AVAILABILITY STATEMENT

The supplementary material for this article can be found online. The data that support the findings of this study are available from the corresponding author upon reasonable request.

## Supporting information

Supplementary Material
