# Supplementary material for: LRRFIP1, an epigenetically regulated gene, is a prognostic biomarker and predicts malignant phenotypes of glioma
Source: CNS Neurosci Ther. 2022 Mar 26;28(6):873–83. doi: 10.1111/cns.13817 (PMC9062568; doi:10.1111/cns.13817)
Supplement: Supplementary file 1 — Supplementary Material [file CNS-28-873-s001.docx]

**
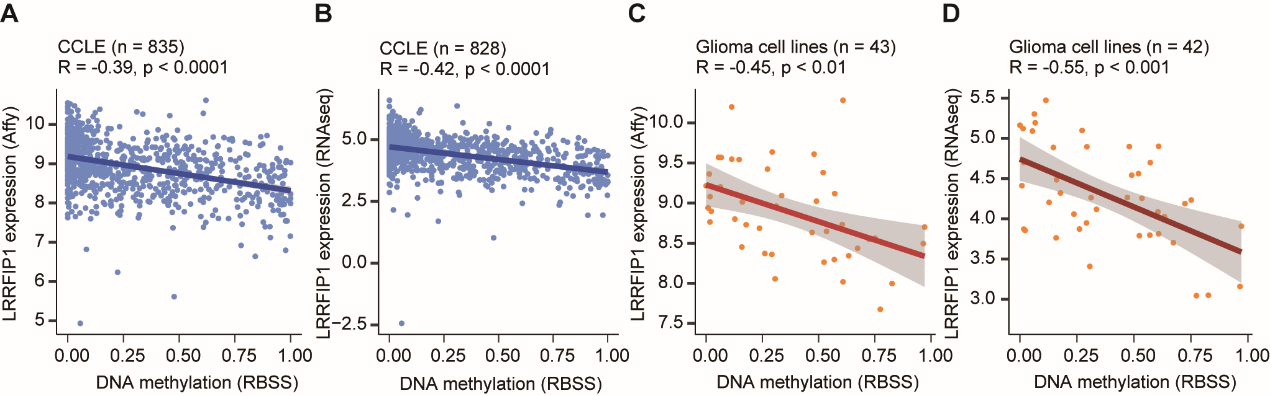
**

**Supplementary FIGURE 1**. Validation of the correlation between the mRNA expression level of *LRRFIP1* and the DNA methylation levels in CCLE and glioma cell lines by Pearson correlation analysis. (A, B) The *LRRFIP1* expression was negatively associated with the DNA methylation level in CCLE. (A: n = 835, R = -0.39, p < 0.0001; B: n = 828, R = -0.41, p < 0.0001.) (C, D) The *LRRFIP1* expression was negatively correlated with the DNA methylation level in glioma cell lines. (C: n = 43, R = -0.45, p < 0.01；n = 42, R = -0.55, p < 0.001.)

**
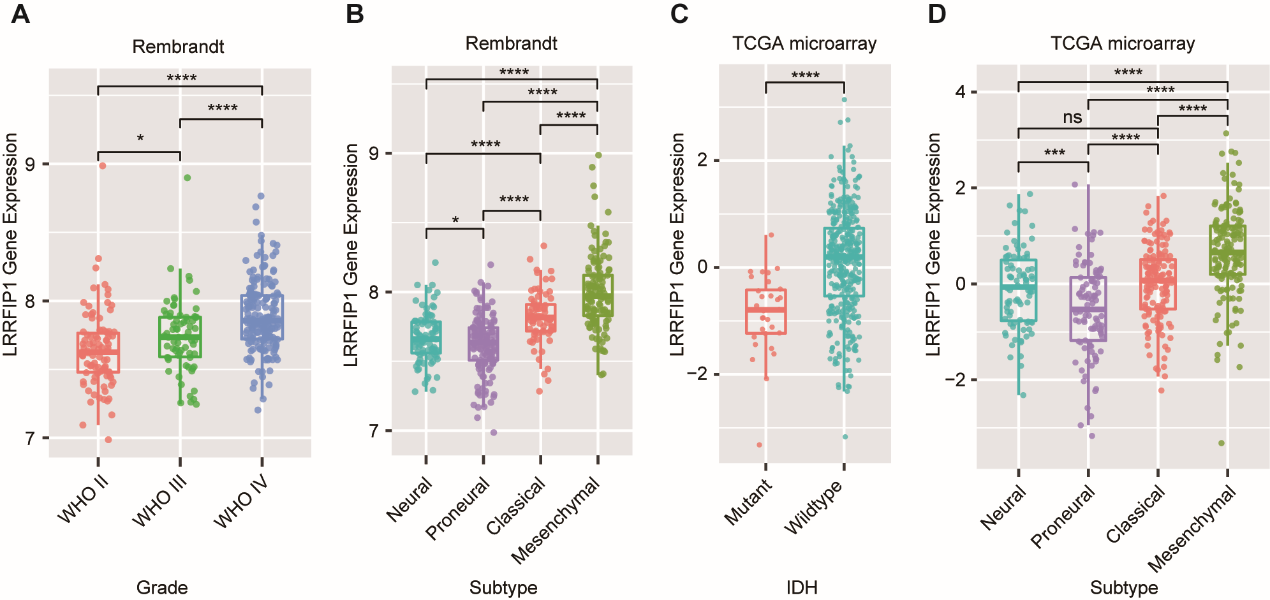
**

**Supplementary FIGURE 2**. *LRRFIP1* mRNA expression pattern in Rembrandt and TCGA microarray. (A) *LRRFIP1* is enriched in high grade glioma in Rembrandt. (B) *LRRFIP1* is enriched in mesenchymal molecular subtype gliomas in Rembrandt. (C) *LRRFIP1* is enriched in *IDH* wildtype gliomas in TCGA microarray. (D) *LRRFIP1* is enriched in mesenchymal molecular subtype gliomas in TCGA microarray. *p < 0.05, ***p < 0.001, ****p < 0.0001.

**
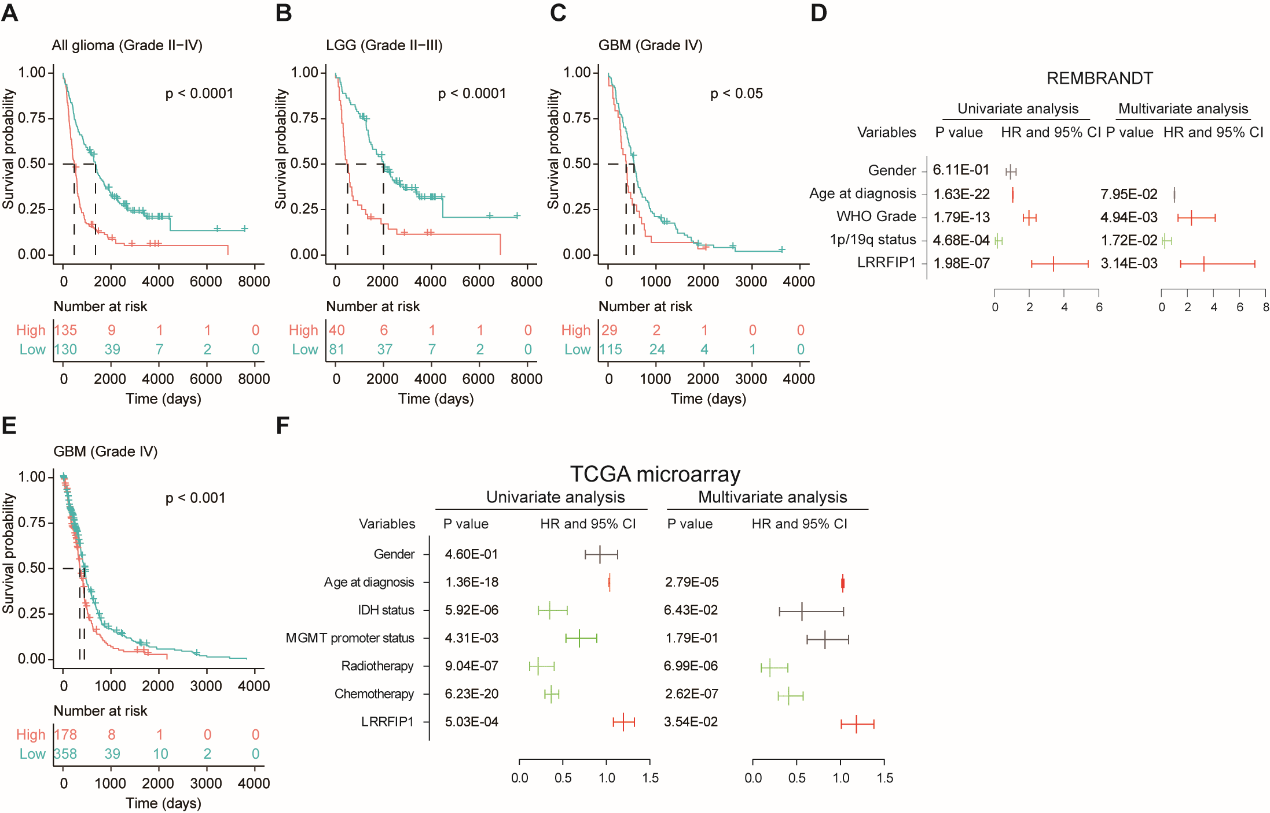
**

**Supplementary FIGURE 3**. *LRRFIP1* mRNA expression was related to clinical outcomes in glioma. (A-C) Kaplan-Meier analysis of the survival of all gliomas, LGG and GBM patients from Rembrandt. (D) Univariate analysis and multivariate analysis were performed for the overall survival in the Rembrandt. (E) Kaplan-Meier analysis of the survival of GBM basis on the TCGA microarray. (F) Univariate analysis and multivariate analysis were performed for the overall survival in the TCGA microarray.

**
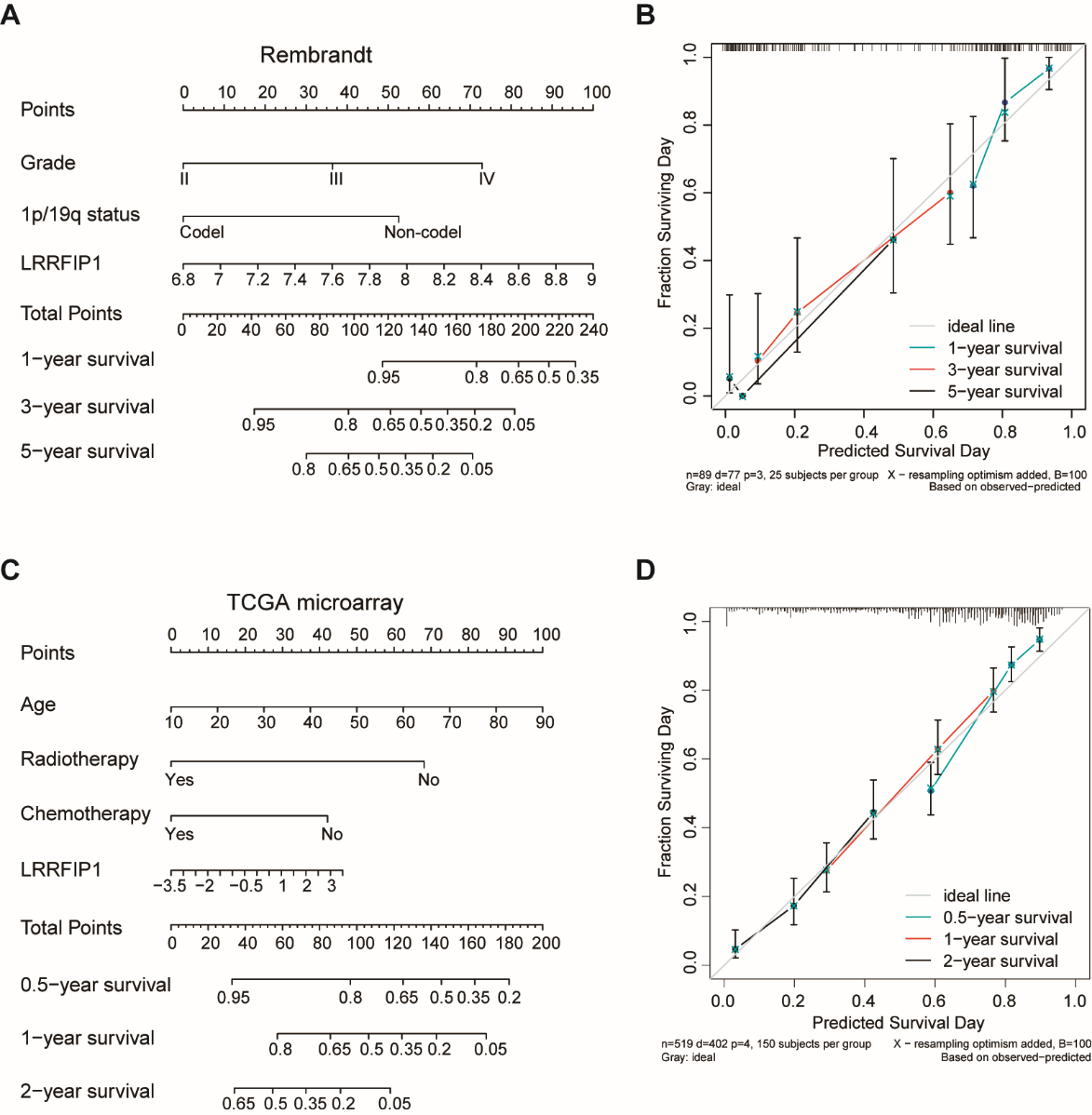
**

**Supplementary FIGURE 4.** Construction and evaluation of the nomogram for predicting overall survival. (A) Nomogram for predicting 1, 3, or 5- year survival in glioma patients, based on the data from Rembrandt. (B) Calibration curves for predicting patient survival at 1, 3, 5 years in Rembrandt. (C) Nomogram for predicting 0.5, 1, or 2- year survival in glioma patients, based on the data from TCGA microarray. (D) Calibration curves for predicting patient survival at 0.5, 1, 2 years in the dataset from TCGA microarray.


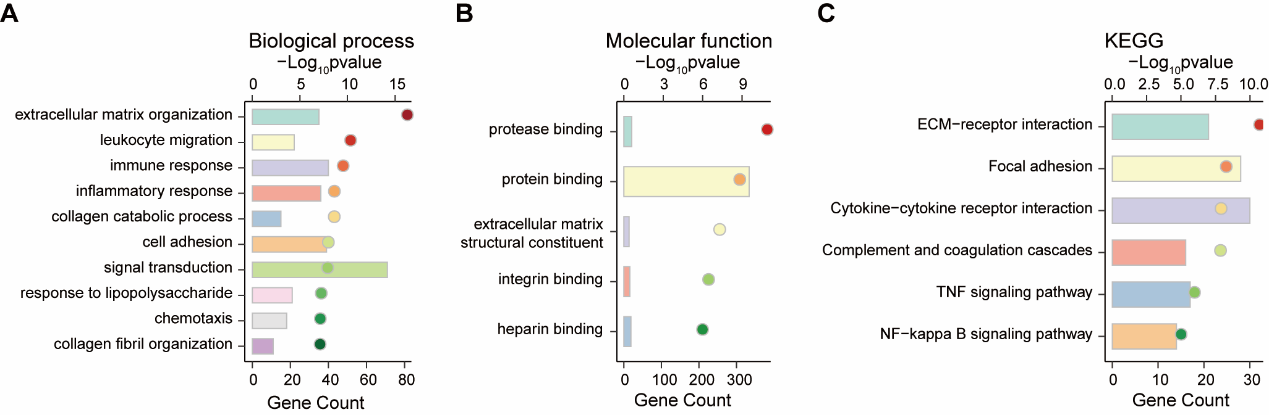


**Supplementary FIGURE 5** The GO and KEGG enrichment analysis of *LRRFIP1* correlated genes in TCGA dataset. (A). *LRRFIP1*-associated biological process in gliomas. (B) *LRRFIP1*-related molecular function in gliomas. (C) KEGG pathways regulated by *LRRFIP1* in gliomas.

**Supplementary table 1** 93 significant methylation genes in glioblastomas

| Gene | HR | 95% CI | | P value |
| --- | --- | --- | --- | --- |
|  |  | Low | High |  |
| TMEM33 | 2.034791 | 1.288009 | 3.214554 | 2.33E-03 |
| ETF1 | 1.870336 | 1.244353 | 2.811226 | 2.60E-03 |
| TXNDC15 | 1.869173 | 1.404752 | 2.487135 | 1.77E-05 |
| ZMYM6 | 1.70261 | 1.183449 | 2.44952 | 4.14E-03 |
| LRRFIP1 | 1.646843 | 1.154756 | 2.348627 | 5.88E-03 |
| VKORC1L1 | 1.613586 | 1.244747 | 2.091718 | 3.02E-04 |
| SLC35D1 | 1.565687 | 1.251594 | 1.958602 | 8.69E-05 |
| RAB1A | 1.557586 | 1.132678 | 2.141893 | 6.40E-03 |
| SNX2 | 1.542425 | 1.103473 | 2.155988 | 1.12E-02 |
| KDELR2 | 1.536492 | 1.213377 | 1.945652 | 3.63E-04 |
| PTPRJ | 1.534519 | 1.144439 | 2.057557 | 4.22E-03 |
| CD63 | 1.492782 | 1.201615 | 1.854503 | 2.96E-04 |
| FN3KRP | 1.492054 | 1.043308 | 2.133815 | 2.84E-02 |
| SPPL2A | 1.473941 | 1.141209 | 1.903685 | 2.96E-03 |
| YIPF4 | 1.473005 | 1.037253 | 2.091817 | 3.04E-02 |
| LAP3 | 1.471293 | 1.206034 | 1.794894 | 1.41E-04 |
| RAP1A | 1.449962 | 1.122934 | 1.872229 | 4.39E-03 |
| SLC36A4 | 1.445256 | 1.123862 | 1.858559 | 4.11E-03 |
| TTC26 | 1.442588 | 1.119879 | 1.858291 | 4.56E-03 |
| C11orf58 | 1.436753 | 1.040635 | 1.983654 | 2.77E-02 |
| FECH | 1.417031 | 1.049026 | 1.914134 | 2.31E-02 |
| PIGB | 1.414027 | 1.115841 | 1.791897 | 4.14E-03 |
| RALB | 1.409481 | 1.136473 | 1.748071 | 1.78E-03 |
| C7orf25 | 1.405602 | 1.088544 | 1.815008 | 9.04E-03 |
| RPE | 1.395656 | 1.019356 | 1.910868 | 3.76E-02 |
| PHTF1 | 1.390549 | 1.089538 | 1.774722 | 8.07E-03 |
| CLDN12 | 1.381526 | 1.068887 | 1.785608 | 1.36E-02 |
| BAZ1B | 1.368926 | 1.069289 | 1.752527 | 1.27E-02 |
| ABHD11 | 1.360412 | 1.025075 | 1.805449 | 3.31E-02 |
| DNMT3A | 1.35888 | 1.080278 | 1.709333 | 8.80E-03 |
| FKBP9 | 1.357961 | 1.145163 | 1.610301 | 4.34E-04 |
| SOCS5 | 1.357517 | 1.024619 | 1.798574 | 3.32E-02 |
| STAM2 | 1.356063 | 1.015217 | 1.811344 | 3.92E-02 |
| APLP2 | 1.348759 | 1.0703 | 1.699664 | 1.12E-02 |
| COQ10B | 1.340363 | 1.05532 | 1.702398 | 1.63E-02 |
| CD276 | 1.324542 | 1.003766 | 1.747829 | 4.70E-02 |
| RNF145 | 1.302602 | 1.027247 | 1.651767 | 2.91E-02 |
| CMTM3 | 1.301592 | 1.014023 | 1.670713 | 3.85E-02 |
| CD44 | 1.295498 | 1.114498 | 1.505895 | 7.47E-04 |
| YKT6 | 1.288566 | 1.015987 | 1.634274 | 3.65E-02 |
| PQLC3 | 1.288133 | 1.102215 | 1.505411 | 1.45E-03 |
| PDGFC | 1.284989 | 1.0788 | 1.530587 | 4.96E-03 |
| SIL1 | 1.276083 | 1.001712 | 1.625605 | 4.84E-02 |
| FSIP1 | 1.272947 | 1.086084 | 1.491962 | 2.89E-03 |
| SLC35F5 | 1.270827 | 1.019602 | 1.583952 | 3.29E-02 |
| TMEM30A | 1.270312 | 1.009138 | 1.59908 | 4.16E-02 |
| ITGAV | 1.268697 | 1.076203 | 1.495621 | 4.59E-03 |
| PKD2 | 1.263575 | 1.042946 | 1.530877 | 1.69E-02 |
| MOBKL1B | 1.261946 | 1.020423 | 1.560635 | 3.18E-02 |
| TMEM9B | 1.260286 | 1.005598 | 1.579478 | 4.46E-02 |
| SLC20A1 | 1.259716 | 1.065797 | 1.488917 | 6.79E-03 |
| C1QTNF9 | 1.251352 | 1.005848 | 1.556777 | 4.42E-02 |
| CYB5R1 | 1.240636 | 1.020796 | 1.507822 | 3.03E-02 |
| CTSB | 1.223545 | 1.059428 | 1.413086 | 6.04E-03 |
| NT5E | 1.178029 | 1.019943 | 1.360618 | 2.58E-02 |
| NRCAM | 1.17397 | 1.011413 | 1.362654 | 3.49E-02 |
| ARNTL | 1.173548 | 1.007767 | 1.3666 | 3.94E-02 |
| LZTS1 | 1.149765 | 1.00538 | 1.314884 | 4.15E-02 |
| AKAP12 | 1.144163 | 1.022086 | 1.280821 | 1.93E-02 |
| RARRES2 | 1.141515 | 1.055628 | 1.23439 | 9.12E-04 |
| DDIT3 | 1.1359 | 1.01026 | 1.277165 | 3.31E-02 |
| RNASE3 | 1.131576 | 1.008214 | 1.270031 | 3.58E-02 |
| SPRY4 | 1.127621 | 1.009196 | 1.259943 | 3.39E-02 |
| GPR65 | 1.104506 | 1.007479 | 1.210877 | 3.41E-02 |
| SCG2 | 1.103396 | 1.005309 | 1.211053 | 3.83E-02 |
| GLIS1 | 0.868128 | 0.754698 | 0.998606 | 4.78E-02 |
| PSTK | 0.829338 | 0.687879 | 0.999887 | 4.99E-02 |
| ZNF488 | 0.809237 | 0.699699 | 0.935923 | 4.34E-03 |
| GNAL | 0.79044 | 0.647534 | 0.964885 | 2.08E-02 |
| TCF15 | 0.786356 | 0.663332 | 0.932196 | 5.63E-03 |
| NKD1 | 0.779141 | 0.670478 | 0.905416 | 1.13E-03 |
| GRHL3 | 0.775899 | 0.672192 | 0.895607 | 5.28E-04 |
| SRPK1 | 0.77514 | 0.618232 | 0.97187 | 2.73E-02 |
| SIX4 | 0.757883 | 0.610368 | 0.94105 | 1.21E-02 |
| RAB26 | 0.746192 | 0.58514 | 0.951572 | 1.83E-02 |
| C3orf59 | 0.740552 | 0.562972 | 0.974147 | 3.18E-02 |
| ILF2 | 0.731836 | 0.552113 | 0.970064 | 2.99E-02 |
| AJAP1 | 0.706638 | 0.541194 | 0.922658 | 1.07E-02 |
| KIF22 | 0.689714 | 0.52328 | 0.909084 | 8.38E-03 |
| HDAC4 | 0.68894 | 0.47599 | 0.997159 | 4.83E-02 |
| TAF1C | 0.663465 | 0.494077 | 0.890925 | 6.37E-03 |
| MED26 | 0.641003 | 0.422805 | 0.971805 | 3.62E-02 |
| SMPD3 | 0.622438 | 0.447341 | 0.866072 | 4.91E-03 |
| PRL | 0.615068 | 0.448621 | 0.843269 | 2.54E-03 |
| CCDC141 | 0.612317 | 0.47476 | 0.789729 | 1.58E-04 |
| ALDOB | 0.608779 | 0.404353 | 0.916554 | 1.74E-02 |
| TCTE1 | 0.605952 | 0.396412 | 0.926252 | 2.07E-02 |
| PCBP3 | 0.57974 | 0.431565 | 0.778791 | 2.94E-04 |
| RBM17 | 0.550457 | 0.411594 | 0.736171 | 5.70E-05 |
| PPP2R5D | 0.545817 | 0.379219 | 0.785604 | 1.12E-03 |
| LMX1A | 0.53657 | 0.290358 | 0.991561 | 4.69E-02 |
| KLK5 | 0.485601 | 0.280217 | 0.841522 | 1.00E-02 |
| PLCXD2 | 0.463934 | 0.265165 | 0.811701 | 7.13E-03 |
